# A Cancer-Indicative microRNA Pattern in Normal Prostate Tissue

**DOI:** 10.3390/ijms14035239

**Published:** 2013-03-04

**Authors:** Olaf J. C. Hellwinkel, Christina Sellier, Yu-Mi Jessica Sylvester, Jan C. Brase, Hendrik Isbarn, Andreas Erbersdobler, Thomas Steuber, Holger Sültmann, Thorsten Schlomm, Christina Wagner

**Affiliations:** 1 Department of Legal Medicine, University Medical Center Hamburg-Eppendorf, Hamburg 20246, Germany; 2 Martini-Clinic, Prostate Cancer Center, University Medical Center Hamburg-Eppendorf, Hamburg 20246, Germany; E-Mails: c.sellier@gmx.de (C.S.); yumisylvester@yahoo.de (Y.-M.J.S.); hendrikisbarn@googlemail.com (H.I.); steuber@uke.uni-hamburg.de (T.St.); Thorsten.Schlomm@uke-hh.de (T.Sc.); cwagner@fz-borstel.de (C.W.); 3 Cancer Genome Research Group, Division of Molecular Genetics, German Cancer Research Center (DKFZ) and National Center for Tumor Diseases, Heidelberg 69120, Germany; E-Mails: brase@sividon.com (J.C.B.); h.sueltmann@dkfz-heidelberg.de (H.S.); 4 Department of Pathology, Medical Center, University of Rostock, Rostock 18055, Germany; E-Mail: andreas.erbersdobler@med.uni-rostock.de

**Keywords:** prostate carcinoma, tumor neighboring normal tissue, microRNA, molecular marker, early neoplastic transformation, field cancerization

## Abstract

We analyzed the levels of selected micro-RNAs in normal prostate tissue to assess their potential to indicate tumor foci elsewhere in the prostate. Histologically normal prostate tissue samples from 31 prostate cancer patients and two cancer negative control groups with either unsuspicious or elevated prostate specific antigen (PSA) levels (14 and 17 individuals, respectively) were analyzed. Based on the expression analysis of 157 microRNAs in a pool of prostate tissue samples and information from data bases/literature, we selected eight microRNAs for quantification by real-time polymerase chain reactions (RT-PCRs). Selected miRNAs were analyzed in histologically tumor-free biopsy samples from patients and healthy controls. We identified seven microRNAs (miR-124a, miR-146a & b, miR-185, miR-16 and let-7a & b), which displayed significant differential expression in normal prostate tissue from men with prostate cancer compared to both cancer negative control groups. Four microRNAs (miR-185, miR-16 and let-7a and let-7b) remained to significantly discriminate normal tissues from prostate cancer patients from those of the cancer negative control group with elevated PSA levels. The transcript levels of these microRNAs were highly indicative for the presence of cancer in the prostates, independently of the PSA level. Our results suggest a microRNA-pattern in histologically normal prostate tissue, indicating prostate cancer elsewhere in the organ.

## 1. Introduction

Understanding the biology of early tumors is of eminent strategic importance, due to its potential impact on the diagnosis, treatment and prognosis of prostate carcinoma. To date, knowledge about early prostate cancer development, however, is still limited. Nevertheless, it is clear that morphologically detectable malignant changes are preceded by aberrations at the molecular-biological level. The volume of such molecularly altered areas should be substantially larger than the histologically manifest cancer or even the prostatic intraepithelial neoplasia (PIN) area therein [1]. These genetic alterations in histologically normal epithelia near tumors, as well as stromal cells in epithelial tumors, have been discussed as “field cancerization” [2].

The identification of such molecular alterations in unsuspicious prostate tissue could lead to an interesting application in clinical routine: the assessment of prostate cancer risks by evaluation of apparently tumor-free biopsy specimens. To date, the definitive diagnosis of prostate cancer is exclusively dependent on prostate biopsies, which are taken from patients with elevated PSA levels (therefore, cancer-suspicious) and examined histopathologically. The absence of malignant cells precludes the diagnosis of cancer. Now, small tumors are often missed by prostate biopsies. In such apparently negative cases, histologically normal biopsy specimens possessing molecular signatures, which suggest the presence of adjacent cancer or imminent progression towards malignancy [2], could represent a diagnostic tool to identify patients at risk of having prostate cancer (on the other side, the determination of an unsuspicious molecular environment could prevent over-diagnostics by repeated biopsy series). Indeed, in an earlier study [3], we identified a transcription signature of five genes in histologically normal prostate tissues, which indicated the presence of neighboring prostate cancer foci.

Only one decade ago, microRNAs—small RNA molecules of 20–22 nucleotides length—have been identified to be important regulators of the human transcriptome associated with several disorders— especially cancer [4,5]. Each microRNA binds to several target transcripts, leading to direct transcript degradation or hindering the translation initiation. A limited number of microRNAs is thus capable of controlling a large number of transcripts [6]. In consequence, miRNA expression signatures could play a significant role in prostate cancer initiation, indicating (pre-)malignant processes within the prostate.

Here, we conducted a pilot study on the expression of well defined microRNAs in normal prostate tissue to test their potential to indicate occult prostate cancer.

## 2. Results and Discussion

### 2.1. A Number of microRNAs Discriminate Tumor-Positive from Tumor-Negative Groups

**Figure 1:**
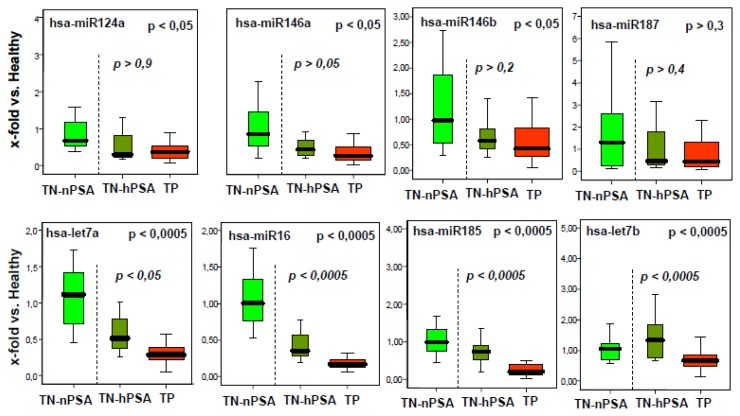
Boxplots (25% and 75% quantiles) for microRNA expression (x-fold transcription compared to the healthy control group, based on relative quantification (RQ) values) in histologically normal tissues in the tumor negative tumor negative/normal PSA (TN-nPSA) (green) and TN-hPSA (olive) groups and the tumor positive tumor-positive (TP) group (red), as determined by the main experiment. *p*-values for microRNA level differences between the three groups are shown in the upper right part of the graphs. *p*-values for microRNA level differences between the diagnostically relevant tumor-negative/high PSA group (TN-hPSA) group and the TP group are given in italic letters.

As for transcripts in our previous study [3], this study was designed to scrutinize the concept of tumor indicative microRNAs in histologically normal tissue from prostate cancer patients. After an initial experimental and logic selection process on the 157 microRNAs with the earliest reports in literature [for details, see experimental section, subheading 3.1 (below)], we decided to test the levels of miR-124a, miR-146a, miR-146b, miR-185, miR-16, let-7a and let-7b in histologically normal prostate biopsies from the tumor-positive group (TP group) compared to both tumor-negative control collectives [tumor-negative/high PSA group (TN-hPSA) group and tumor negative/normal PSA (TN-nPSA) group; see Table 1 for the description of the groups]. The results are shown in Figure 1.

Indeed, we found a downregulation of these microRNAs in the tumor positive group. As demonstrated by explorative univariate statistics on three groups (Kruskal-Wallis tests), the differences between these collectives were significant (see upper *p*-values in the graphs in Figure 1). miR-187 (a microRNA designed as the control, as it was expressed in our prostate tissue pool, but not fitting to the selection criteria described above) was not found to be differentially expressed. The dCt values of the internal standards RNU-44 and -48 were similar between all groups, indicating even source RNA quantities in the investigated biopsies (supplemental figure, upper part).

Interestingly, the selected microRNAs were all found to be downregulated in cancer-free biopsies from patients with prostate cancer (TP group) compared to both age-matched cancer negative groups (TN-nPSA group and TN-hPSA group). This could indicate that the shutdown of these microRNAs is involved in early malignant transformation (see below). If this is true, it would fit with observations of an extensive micro-transcriptome repression in “mature” cancer tissues [7,8], indicating a role of most microRNAs in tissue differentiation or tumor suppression. Alternatively, it may be speculated that the microRNA signature found here could represent a physiological or enforced reaction of normal cells and tissues to neighboring tumor foci. In any case, reduced transcription patterns of miR-124a, miR-146a and b, miR-185, miR-16 and let-7a and b could be considered as indicators of occult tumors in the vicinity.

An attempt to find a robust marker set indicating occult prostate cancer, however, should regard the clinical everyday situation. There, usually only individuals with high PSA values are submitted to intensive diagnostic procedures, such as biopsy-taking. Therefore, we tested whether the observed differential microRNA transcription pattern discriminates the TP group from the TN-hPSA group alone (both groups with elevated PSA levels; see Table 1). In this comparison, levels of four microRNAs—let-7a, let-7b, miR-16 and miR-185—remained to be significantly different, as shown by explorative univariate statistics on two groups (Mann-Whitney U tests; see lower *p*-values in the graphs in Figure 1).

The quantification of gene markers for epithelial (*ARG, GUSB*) and stromal (*COL1A1*) tissue resulted in comparable gene expression rates in the TP group and the TN-hPSA group (supplemental figure, lower part), suggesting that the proportions of different tissue types in the biopsies were similar.

### 2.2. The miRNA Expression Signature Discriminates the Tumor-Positive from Tumor-Negative Groups Better than Single microRNA Levels

**Figure 2:**
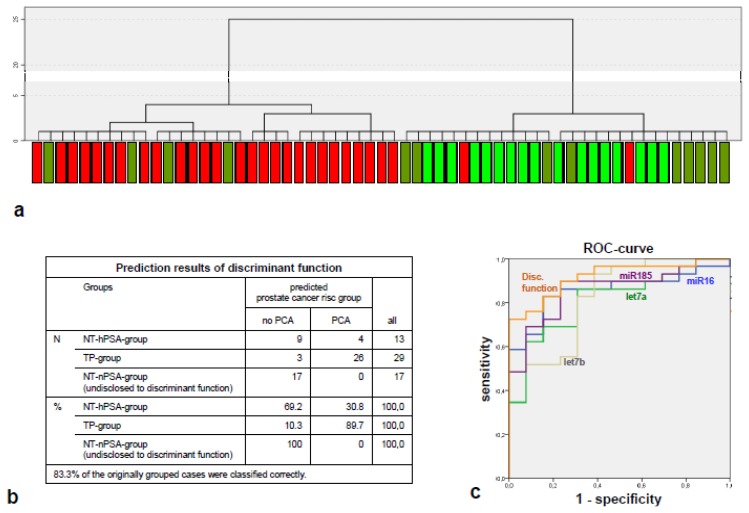
Results of the multivariate analyses on the main experiment. (**a**) Unsupervised cluster analysis generated on the expression levels of let-7a, let-7b, miR-16 and miR-185. Single cases are labeled as green, olive or red depending on their group affiliation (TN-nPSA group, TN-hPSA group and TP group respectively); (**b**) Classification results of the discriminant function generated on the expression level signature of let-7a, let-7b, miR-16 and miR-185; (**c**) receiver operating characteristic (ROC) curve for the estimation of sensitivity and specificity of the discriminators let-7a, let-7b, miR-16 and miR-185 and the discriminant function. Note that the area under the curve (AUC) of the signature (represented by the discriminant function) is larger than the AUCs of the single microRNAs.

An unsupervised cluster analysis on 58 samples with full quantification results on miR-185, miR-16 and let-7a and b grouped almost all patients of the TP group (27 of 29) in one principal cluster, dividing it from a second principal cluster composed of individuals from both TN groups (Figure 2a).

A discriminant function assigned almost 90% of the TP group individuals correctly to suffer from PCa, while 70% of the TN-hPSA group was correctly “diagnosed” to be tumor free. Notably, 100% of the TN-nPSA group (which originally was not considered for the creation of the discriminant function and, therefore, fulfils the criteria of a test collective), was correctly assigned to be tumor-free. Overall, 83.3% of all individuals were correctly classified (Figure 2b).

The putative sensitivity and specificity of the microRNA signature for the identification of the diagnostically relevant groups (TP group *vs*. TN-hPSA group) was examined by a ROC curve (Figure 2c) and reached 91.8% of area under the ROC curve, exceeding the most informative univariate predictor, namely miR-185 (85.9%; although this particular observation was not tested for significance).

### 2.3. The Components of the Tumor-Indicative microRNA Signature Have Tumor-Suppressive Properties in Other Tumors

let-7a, let-7b, miR-16 and miR-185 are known to regulate tumorigenic processes in general or in prostate cancer. Let-7a has been shown to downregulate the transcript of the *MYC* oncogene in Burkitt lymphoma cells [9] and in laryngeal cancer [10]. Elevated expression of *MYC* is a central event in the early development of human malignancies. Low level overexpression of *MYC* in mouse models leads to the development of PIN-like epithelial cell abnormalities [11] and results in the development of prostate cancer [12]. Previously, we could demonstrate *MYC* to be upregulated within the same collective (TP group [3])—a causal relationship of these findings with the let-7a repression demonstrated here might be supposed [indeed, we found a weak correlation between *MYC* upregulation and let7a repression in dCt value cross-comparisons—however, it did not reach our significance criteria (not shown)]. It also has been shown that let-7a downregulates the oncogenes, *E2F2* and *cyclin D2* [13]. Let-7b is targeting important cell cycle molecules in malignant melanoma cells and interferes with anchorage-independent growth [14]. miR-16 has been found to be downregulated in prostate cancer [15]. Also, miR-16 controls prostate cancer [16] and non-small cell lung cancer [17], together with miR-15a, by targeting and downregulating multiple oncogenic activities. It also has been shown that both microRNAs induce apoptosis by targeting Bcl2 [18]. Finally, miR-185 was shown to induce cell cycle arrest in human non-small cell lung cancer cell lines [19]. These studies demonstrate evidently the tumor suppressive functions of these four microRNAs. Their downregulation supports models suggesting a stepwise accumulation of early, oncogenic molecular changes in premalignant cells during carcinogenesis [2,20–23].

## 3. Experimental Section

### 3.1. Pre-Selection of microRNAs

To test principal miRNAs expression in prostate tissue, we prepared an RNA pool of tumor negative prostate biopsies from 17 healthy donors (Table 1, supplementary Table 1) to assess the expression of 157 microRNAs by quantitative RT-PCRs. Ninety four of 154 microRNAs were found to be expressed in prostate tissue (showing regular, sigmoid shaped PCR curves of both technical replicates at a dCt value dissimilarity ≤1; see supplementary Table 2). On these expressed microRNAs, we applied the following selection tree on the miRó microRNA database and PubMed inquiries to define PCa marker candidates:

1. Are the microRNAs associated with prostate cancer (according to MiRó data)? If yes, then:
2. Are (prostate-) cancer-related target transcripts of the microRNAs experimentally validated or else consistently predicted by three independent prediction algorithms (TargetScan, miRanda and PicTar)? If yes, then:
3. Are there reports on a role in human early cancer formation, malignant transformation or field cancerization in scientific literature (PubMed search results up to the year 2008)?

The following microRNAs fitted best to these requirements and were chosen for further analyses in the main experiment: miR-124a, miR-146a, miR-146b, miR-185, miR-16, let-7a and let-7b.

miR-187—a microRNA expressed in our prostate tissue pool, but not fitting to the selection criteria described above—was also included to the main experiment analysis for control purposes (see below).

### 3.2. Collectives Analyzed in the Main Experiment

For the main experiment, we defined three collectives of tumor-free prostate biopsies taken by diagnostic ultrasound-guided transrectal biopsy probing (Table 1; detailed data in supplementary Table 1):

1. the tumor-positive group (TP group) composed of patients with proven PCa,
2. the tumor negative/high PSA group (TN-hPSA group), composed of patients with an elevated PSA level, but a high likelihood of being cancer-free (as confirmed by multiple repeated negative biopsies [24]),
3. the tumor negative/normal PSA group (TN-nPSA group), comprising healthy men with normal serum PSA (<1 ng/mL).

### 3.3. Tissue Sample Acquisition and RNA Isolation

The approval for the study was obtained from the local ethical committee. All patients gave written permission to additional tissue sampling for scientific purposes.

Tissue specimens from all individuals were collected as previously described [3]. Shortly, all tissue samples were obtained from the peripheral zone of the prostate by transrectal prostate biopsies and immediately fixed in RNAlater (Qiagen, Hilden, Germany). HE staining of representative longitudinal cryo-sections was done to ensure the absence of cancer or PIN. Only tissues that contained between 30% and 50% epithelia were enrolled into further analysis. RNA was isolated from the remaining cryo-samples using RNeasy columns (Qiagen), following an adapted protocol for separate isolation of RNA and microRNA (the flow-throughs from the first washing step along with microRNA and small RNA fragments therein were preserved, mixed with 3 × vol 100% EthOH and administrated on new RNeasy columns; the following usual washing steps were done as described by Qiagen). MicroRNA quantity and quality was assessed using PicoRNA kits and an Agilent 2100 bioanalyzer (Agilent Technologies, Waldbronn, Germany; measuring the integral of the peak at 20 to 50 nucleotides) and the NanoDrop technology (NanoDrop products; Wilmington, DE, USA).

### 3.4. Quantitative RT-PCR

20 ng of microRNA fractions were reversely transcribed to cDNA in volumes of 20 μL using the cDNA archive kit, according to the manufacturer’s protocol (Applied-Biosystems, Darmstadt, Germany), using specific loop-RT-primers for all investigated mature microRNAs (see below). RT-PCRs were performed on the Gene-analyzer 7900 (Applied Biosystems) using microRNA assays on demand (Applied Biosystems; pre-designed primer-pair and Taq-man probe combinations) to amplify up to 157 microRNAs (supplementary Table 2) in duplicates, according to the manufacturer’s guidelines.

For initial screens in the healthy prostate tissue pool, RT-PCR runs were examined for principal microRNA expression by assessment of dCt values and regular sigmoid shaped curves. For the main experiment, relative expression differences of selected microRNAs between the analyzed collectives were calculated using the ΔΔCt method [25]. Small nuclear RNAs, RNU44 and RNU48, were applied as internal standards. Mean target microRNA ΔCt values of the healthy control collective (TN-nPSA group) were defined as calibrators; resulting RQ values (relative quantification) on all microRNAs have to be understood as x-fold expressions compared to mean expressions in the TN-nPSA group.

Levels of tissue type-specific transcripts for epithelial (*ARG*, *GUSB*) and stromal (*COL1A1*) cells were analyzed on all groups by RT-PCRs, as previously published [3], to test the biopsies for similar distributions of tissue types.

### 3.5. Statistical Evaluation

Due to non-Gaussian distribution patterns of the results, non-parametric tests (Mann-Whitney U and Kruskal-Wallis tests) were applied to test for significant microRNA level differences of the compared groups. Transcript level differences were considered to be significant when means of the RNU44/48-double-normalized RQ sets displayed a *p*-value <0.05. Additional analyses implied a non-supervised cluster analysis (Ward method on squared Euclidic distances) and an analysis for the creation of a discriminant function on the TP group and the TN-hPSA group, implying all individuals with complete quantification results of the included microRNAs. Sensitivities and specificities were compared by ROC curves. Statistic evaluation was performed using Microsoft Excel and SPSS.

## 4. Conclusions

In our previous study, we found indications for the existence of a gene transcription phenotype in histologically normal prostate tissue, indicating neighboring tumors [3]. Here, the identification of a microRNA signature with similar properties expands these possibilities, as tests on microRNAs have some methodological advantages compared to mRNAs (microRNAs are less sensitive to tissue degradation [26] and fixation and are degraded less rapidly—microRNA tests, thus, could be more successful, even after formalin fixation and/or after prolonged storage of prostate tissues). Additionally, due to their diminutive length, microRNAs diffuse easily through cellular and tissue barriers, leading to organ-wide extracellular microRNA levels, which could reflect particular microRNA signatures in dispersed tumor foci. In this way, the local measurement of cancer-typical microRNA patterns in unsuspicious tissue compartments could indicate tumors in the vicinity.

However, our study has some limitations. First, the selection procedure explained in the experimental section missed some miRNAs with a high consensus in newer prostate cancer research, as miR-96, miR-221/222 or miR-205 (miRNAs described as deregulated in mature PCa compared to normal tissues in most profiling studies [27–30]). Hence, comprehensive array screenings are required to obtain inclusive information about the micro-transcriptome in premalignant prostate tissues. Second, our analysis has not been validated on independent collectives so far. It consequently has to be viewed as a pilot study. Finally, studies are lacking that could elucidate the functional role of the studied microRNAs in prostate carcinogenesis.

The clear, significant microRNA level differences found between the compared groups nevertheless give reason to further test our microRNA signature on larger and independent collectives for their applicability as diagnostic surrogate markers in biopsies from patients with elevated PSA serum levels.

## Figures and Tables

**Table 1:** Overview on the analyzed tissue collectives.

Prostate biopsies with normal, tumor free tissue (30% to 50% epithelia) from			
Groups	Healthy men (tumor negative)	Healthy men (with initial suspicion for prostate cancer (PCa), but found to be tumor negative)	PCa patients (with proven malignant prostate tumor)
Number of individuals	*n* = 17	*n* = 14	*n* = 31
Years of age (10%- & 90%-quantiles)	51 to 64	54 to 75	54 to 66
Diagnostics	negative palpation, negative diagnostic biopsy series	negative or unclear palpation, multiple negative diagnostic biopsy series	unclear or positive palpation, ≥one tumor positive diagnostic biopsy
	low prostate specific antigen (PSA) level (mean 0.78 ng/mL [±0.26 SD])	elevated PSA level (mean 6.44 ng/mL [±4.82 SD])	elevated PSA level (mean 7.97 ng/mL [±5.21 SD])
Designations	Tumor-negative/normal PSA group (TN-nPSA group)	Tumor-negative/high PSA group (TN-hPSA group)	Tumor-positive group (TP group)
Note		groups representing men with elevated PSA levels submitted to diagnostic biopsy-taking in clinical routine	

## Supplementary Files

Supplementary File 1 Supplemental Table (XLS, 66 KB)

## References

[1] Albini A., Sporn M.B. (2007). The tumour microenvironment as a target for chemoprevention. Nat. Rev. Cancer.

[2] Dakubo G.D., Jakupciak J.P., Birch-Machin M.A., Parr R.L. (2007). Clinical implications and utility of field cancerization. Cancer Cell. Int.

[3] Schlomm T., Hellwinkel O.J., Buness A., Ruschhaupt M., Lubke A.M., Chun F.K., Simon R., Budaus L., Erbersdobler A., Graefen M. (2009). Molecular cancer phenotype in normal prostate tissue. Eur. Urol.

[4] Alvarez-Garcia I., Miska E.A. (2005). MicroRNA functions in animal development and human disease. Development.

[5] Yue J., Tigyi G. (2006). MicroRNA trafficking and human cancer. Cancer Biol. Ther.

[6] Pillai R.S. (2005). MicroRNA function: Multiple mechanisms for a tiny RNA?. RNA.

[7] Lu J., Getz G., Miska E.A., Alvarez-Saavedra E., Lamb J., Peck D., Sweet-Cordero A., Ebert B.L., Mak R.H., Ferrando A.A. (2005). MicroRNA expression profiles classify human cancers. Nature.

[8] Chang T.C., Yu D., Lee Y.S., Wentzel E.A., Arking D.E., West K.M., Dang C.V., Thomas-Tikhonenko A., Mendell J.T. (2008). Widespread microRNA repression by Myc contributes to tumorigenesis. Nat. Genet.

[9] Sampson V.B., Rong N.H., Han J., Yang Q., Aris V., Soteropoulos P., Petrelli N.J., Dunn S.P., Krueger L.J. (2007). MicroRNA let-7a down-regulates MYC and reverts MYC-induced growth in Burkitt lymphoma cells. Cancer Res.

[10] Long X.B., Sun G.B., Hu S., Liang G.T., Wang N., Zhang X.H., Cao P.P., Zhen H.T., Cui Y.H., Liu Z. (2009). Let-7a microRNA functions as a potential tumor suppressor in human laryngeal cancer. Oncol Rep.

[11] Zhang X., Lee C., Ng P.Y., Rubin M., Shabsigh A., Buttyan R. (2000). Prostatic neoplasia in transgenic mice with prostate-directed overexpression of the c-myc oncoprotein. Prostate.

[12] Ellwood-Yen K., Graeber T.G., Wongvipat J., Iruela-Arispe M.L., Zhang J., Matusik R., Thomas G.V., Sawyers C.L. (2003). Myc-driven murine prostate cancer shares molecular features with human prostate tumors. Cancer Cell.

[13] Dong Q., Meng P., Wang T., Qin W., Wang F., Yuan J., Chen Z., Yang A., Wang H. (2010). MicroRNA let-7a inhibits proliferation of human prostate cancer cells in vitro and in vivo by targeting E2F2 and CCND2. PLoS One.

[14] Schultz J., Lorenz P., Gross G., Ibrahim S., Kunz M. (2008). MicroRNA let-7b targets important cell cycle molecules in malignant melanoma cells and interferes with anchorage-independent growth. Cell. Res.

[15] Schaefer A., Jung M., Mollenkopf H.J., Wagner I., Stephan C., Jentzmik F., Miller K., Lein M., Kristiansen G., Jung K. (2010). Diagnostic and prognostic implications of microRNA profiling in prostate carcinoma. Int J. Cancer.

[16] Bonci D., Coppola V., Musumeci M., Addario A., Giuffrida R., Memeo L., D’Urso L., Pagliuca A., Biffoni M., Labbaye C. (2008). The miR-15a-miR-16–1 cluster controls prostate cancer by targeting multiple oncogenic activities. Nat. Med.

[17] Bandi N., Zbinden S., Gugger M., Arnold M., Kocher V., Hasan L., Kappeler A., Brunner T., Vassella E. (2009). miR-15a and miR-16 are implicated in cell cycle regulation in a Rb-dependent manner and are frequently deleted or down-regulated in non-small cell lung cancer. Cancer Res.

[18] Cimmino A., Calin G.A., Fabbri M., Iorio M.V., Ferracin M., Shimizu M., Wojcik S.E., Aqeilan R.I., Zupo S., Dono M. (2005). miR-15 and miR-16 induce apoptosis by targeting BCL2. Proc. Natl. Acad. Sci. USA.

[19] Takahashi Y., Forrest A.R., Maeno E., Hashimoto T., Daub C.O., Yasuda J. (2009). MiR-107 and MiR-185 can induce cell cycle arrest in human non small cell lung cancer cell lines. PLoS One.

[20] Braakhuis B.J., Tabor M.P., Kummer J.A., Leemans C.R., Brakenhoff R.H. (2003). A genetic explanation of Slaughter’s concept of field cancerization: Evidence and clinical implications. Cancer Res.

[21] Kakizoe T. (2006). Development and progression of urothelial carcinoma. Cancer Sci.

[22] Roesch-Ely M., Nees M., Karsai S., Ruess A., Bogumil R., Warnken U., Schnolzer M., Dietz A., Plinkert P.K., Hofele C. (2007). Proteomic analysis reveals successive aberrations in protein expression from healthy mucosa to invasive head and neck cancer. Oncogene.

[23] Hockel M., Dornhofer N. (2005). The hydra phenomenon of cancer: Why tumors recur locally after microscopically complete resection. Cancer Res.

[24] Walz J., Graefen M., Chun F.K., Erbersdobler A., Haese A., Steuber T., Schlomm T., Huland H., Karakiewicz P.I. (2006). High incidence of prostate cancer detected by saturation biopsy after previous negative biopsy series. Eur. Urol.

[25] Pfaffl M.W. (2001). A new mathematical model for relative quantification in real-time RT-PCR. Nucleic Acids Res.

[26] Zubakov D., Boersma A.W., Choi Y., van Kuijk P.F., Wiemer E.A., Kayser M. (2010). MicroRNA markers for forensic body fluid identification obtained from microarray screening and quantitative RT-PCR confirmation. Int. J. Legal Med.

[27] Sevli S., Uzumcu A., Solak M., Ittmann M., Ozen M. (2010). The function of microRNAs, small but potent molecules, in human prostate cancer. Prostate Cancer Prostatic Dis.

[28] Coppola V., De Maria R., Bonci D. (2010). MicroRNAs and prostate cancer. Endocr. Relat. Cancer.

[29] Gandellini P., Folini M., Zaffaroni N. (2010). Emerging role of microRNAs in prostate cancer: Implications for personalized medicine. Discov. Med.

[30] Gandellini P., Folini M., Longoni N., Pennati M., Binda M., Colecchia M., Salvioni R., Supino R., Moretti R., Limonta P. (2009). miR-205 Exerts tumor-suppressive functions in human prostate through down-regulation of protein kinase Cepsilon. Cancer Res.

